# Mechanically Enhanced and Reprocessable Vanillin-Based Epoxy Resin via Synergistic Effect of Rigid Cross-Linked Networks and Alkyl Dangling Chains

**DOI:** 10.3390/polym18101226

**Published:** 2026-05-17

**Authors:** Likang Zhou, Songjie Xu, Junhao Fei, Meng Ma, Huiwen He, Yanqin Shi, Yulu Zhu, Si Chen, Xu Wang

**Affiliations:** 1 College of Advanced Materials Engineering, Jiaxing Nanhu University, Jiaxing 314001, China; zhoulikang@jxnhu.edu.cn (L.Z.); fjh240031@jxnhu.edu.cn (J.F.); 2 College of Materials Science and Engineering, Zhejiang University of Technology, Hangzhou 310014, China; xusongjie7890@163.com (S.X.); mameng@zjut.edu.cn (M.M.); hehuiwen@zjut.edu.cn (H.H.); shiyanqin@zjut.edu.cn (Y.S.); zyl311@zjut.edu.cn (Y.Z.); 3 Pinghu Institute of Advanced Materials, Zhejiang University of Technology, Jiaxing 314200, China

**Keywords:** vanillin, Schiff base, alkyl dangling chain, repeated processing, reversible cross-linking

## Abstract

The cross-linked network structure of epoxy resins gives them excellent mechanical properties and heat resistance. However, it also makes them difficult to reprocess and recycle. This leads to environmental pollution and resource waste. Dynamic covalent bonds can make epoxy resins reprocessable. However, this involves a hard trade-off: adding flexible segments improves processing stability at the cost of mechanical strength, whereas keeping a rigid backbone retains the initial strength but leads to incomplete network reformation after multiple reprocessing cycles. As a result, performance continues to decrease. To solve this problem, this paper proposes a new strategy. It combines rigid cross-linked networks with alkyl dangling chains. The strategy does not sacrifice the rigid backbone of the epoxy. Instead, the alkyl dangling chains form physical entanglements during reprocessing. These entanglements compensate for the loss of chemical cross-linking density. Thus, the mechanical properties are retained or even enhanced. A vanillin-based Schiff base epoxy system was used. Alkyl dangling chains of different lengths were compared, and the results show that the system with longer alkyl dangling chains had higher mechanical properties after three reprocessing cycles; its tensile toughness increased by 85.7% compared to the system without dangling chains. At the same time, its thermal stability and glass transition temperature remained almost unchanged. This strategy effectively solves the conflict between strength and processing stability in reprocessable epoxy resins, as well as providing a new idea for designing green, high-performance, and closed-loop recyclable epoxy materials.

## 1. Introduction

Epoxy resins are widely used in coatings, electronic packaging, composites, and adhesives because of their excellent thermal and mechanical properties [1,2,3,4]. However, although the cross-linked networks of epoxy resins give them good chemical stability and solvent resistance, they prevent the material from melting at high temperatures or dissolving in organic solvents. As a result, it is hard to recycle the material efficiently after disposal. Traditional recycling methods, such as pyrolysis or solvolysis [5,6], are not only inefficient but also tend to cause secondary pollution. Among various recycling strategies [7,8,9], direct reprocessing has attracted much attention because of its high efficiency and low pollution [10]. To make epoxy resins reprocessable, researchers have introduced dynamic covalent bonds into the epoxy networks [11,12,13,14,15]. This gives the material both the network structure of thermosets and the processing characteristics of thermoplastics.

Although dynamic covalent bonds provide a feasible way to reprocess epoxy resins, existing systems face a hard trade-off: On the one hand, to improve the ability of networks to rearrange during multiple processing cycles, flexible molecular chains are needed because they increase segmental mobility. However, this significantly reduces the initial mechanical strength of the material and limits use in fields such as composites, which require high strength [16]. On the other hand, keeping the rigid cross-linked network maintains high initial mechanical properties. However, during multiple processing cycles, steric hindrance and side reactions prevent the networks from fully reforming [17,18]. As a result, the mechanical properties continue to decrease. This trade-off is a key bottleneck, as it prevents reprocessable epoxy resins from achieving high performance and practical use. Therefore, there is an urgent need to redesign the epoxy network structure that contains dynamic covalent bonds. The new design should allow the material to retain or even enhance mechanical properties during multiple processing cycles.

To address this problem, existing research has mainly focused on two approaches: one is to adjust the proportion of flexible segments, while the other is to introduce specific dynamic bonds. These methods try to balance strength and processing stability. For example, Mo [19] et al. used flexible polyether amine and rigid isophorone diamine to cure epoxidized vanillin. By adjusting the ratio of the two amines, they obtained epoxy resins with good mechanical properties and reprocessability. Chen [20] et al. used flexible silane chains to prepare aliphatic epoxy resins. They cured these resins with aliphatic dicarboxylic acids, obtaining highly flexible and reprocessable epoxy systems. Jin [21] et al. used dynamic vulcanization to cross-link eugenol-based epoxy resins. The introduction of disulfide bonds gave the epoxy resin a high elongation at break, good flexibility, and reprocessability. Similarly, alternative strategies have been demonstrated to effectively enhance the toughness, thermal stability, and overall mechanical performance of epoxy systems, such as through the incorporation of bio-based modifiers like acacia honey or functionalized nanofillers, such as aminoacetic acid-treated aluminum nitride [22,23]. However, these studies still struggle to maintain high strength while avoiding performance loss after multiple reprocessing cycles.

Inspired by the synergistic reinforcement of physical and chemical cross-linking in thermoplastic elastomers, this study proposes a novel design strategy: constructing a chemically cross-linked backbone using rigid and reversible Schiff base bonds to ensure matrix strength and thermal stability. Meanwhile, alkyl dangling chains of varying lengths are introduced. Unlike previous strategies that sacrifice strength by incorporating flexible segments into the main chain, these do not alter the rigidity of the backbone. The core hypothesis is that during repeated processing, the alkyl dangling chains, which still possess relatively free mobility within the cross-linked network, can form reversible physical entanglements under hot pressing conditions. These entanglements act as transient physical cross-links that compensate for the loss of chemical cross-linking density resulting from incomplete dynamic bond exchange (e.g., side reactions, steric hindrance). In previous studies, this mechanism failed because flexible segments were integrated into the main chain, thereby reducing network rigidity, or because the dangling chains were too short to form entanglements. By maintaining the integrity of the rigid backbone and using sufficiently long alkyl side chains, a synergistic effect is achieved that allows the material to maintain or even enhance its mechanical properties after multiple reprocessing cycles.

## 2. Materials and Methods

### 2.1. Materials

Vanillin (VAN, 99.0%), 1,6-hexanediamine (HDA, ≥99.0%), ethanol (EtOH, ≥99.7%), epichlorohydrin (ECH, 99.0%), tetrabutylammonium bromide (TBAB, 99.0%), sodium hydroxide (NaOH, 96.0%), anhydrous sodium sulfate (Na_2_SO_4_, 99.0%), m-xylylenediamine (MXDA, 99%), Tetrahydrofuran (THF, 99%), butyl glycidyl ether (BGE, >98.0%), and C_12–14_ alkyl glycidyl ether (AGE, 310 g·eq^−1^) were purchased from Shanghai Aladdin Chemical Reagent Co., Ltd. (Shanghai, China). All chemicals were used as received without further purification.

### 2.2. Synthesis of Vanillin-Capped 1,6-Hexanediamine Precursor (VAN-HDA)

Vanillin and 1,6-hexanediamine at a molar ratio of 2:1 were weighed. Vanillin was added into a 500 mL round-bottom flask and dissolved in 200 mL of ethanol. Meanwhile, 1,6-hexanediamine was dissolved in 100 mL of ethanol and placed in a constant-pressure dropping funnel. The diamine solution was added dropwise into the vanillin solution at room temperature under stirring for 1 h, followed by further reaction for 12 h. A yellow precipitate of VAN-HDA precursor formed. After vacuum filtration, the obtained powder was dried in an oven at 60 °C for 24 h to give the VAN-HDA product. Yield: 86.7%.

### 2.3. Synthesis of Vanillin-Based Epoxy Resin (EP-VAN-HDA)

VAN-HDA precursor and epichlorohydrin at a molar ratio of 1:15 were added into a 250 mL three-necked flask equipped with a reflux condenser. Then, 0.1 molar equivalent of TBAB was added as a phase-transfer catalyst. The mixture was heated to 80 °C under nitrogen and then refluxed until VAN-HDA dissolved completely in epichlorohydrin, forming a golden-yellow transparent solution. The solution was then cooled to room temperature. Subsequently, 1.2 molar equivalents of 40 wt% NaOH solution were placed in a constant-pressure dropping funnel and added dropwise into the golden-yellow solution at room temperature over 1 h. The reaction continued for several hours until the pH of the solution became nearly neutral. The obtained suspension was filtered and dried over anhydrous Na_2_SO_4_ to give a clear epoxy solution, which was further concentrated by rotary evaporation to afford a golden-yellow liquid epoxy resin EP-VAN-HDA. Yield: 91.6%.

### 2.4. Preparation of m-Xylylenediamine Curing Agents Grafted with Alkyl Glycidyl Ethers of Different Chain Lengths (MXDA-BGE or MXDA-AGE)

MXDA and BGE (or AGE) were weighed according to a 1:1 molar ratio of MXDA to epoxy groups, as detailed in Table S1. MXDA was placed in a 250 mL single-necked flask and preheated to 80 °C under nitrogen. BGE or AGE was placed in a 100 mL constant-pressure dropping funnel and added dropwise into the MXDA over 1 h, followed by further reaction for 2 h. The grafted product, i.e., MXDA-BGE or MXDA-AGE, was obtained without further purification.

### 2.5. Curing of EP-VAN-HDA with MXDA, MXDA-BGE, or MXDA-AGE

EP-VAN-HDA was weighed with MXDA, MXDA-BGE, or MXDA-AGE according to a 1:1 molar ratio of epoxy groups to active hydrogen, as detailed in Table S2. EP-VAN-HDA was placed in a beaker and mechanically stirred. The curing agent (MXDA, MXDA-BGE, or MXDA-AGE) was added dropwise slowly. After complete addition, the mixture was quickly poured into a silicone mold (10 mm × 10 mm) and cured in an oven at 80 °C or 60 °C for 4 h.

### 2.6. Reprocessing of EP-VAN-HDA/MXDA, EP-VAN-HDA/MXDA-BGE, and EP-VAN-HDA/MXDA-AGE

The pre-cured epoxy samples (EP-VAN-HDA/MXDA, EP-VAN-HDA/MXDA-BGE, and EP-VAN-HDA/MXDA-AGE) were crushed and then hot-pressed at 80–120 °C for 60 min. To further investigate the molding quality and mechanical property changes after repeated hot pressing, the three samples were each crushed into powder and then hot-pressed at 120 °C for 60 min to obtain reprocessed epoxy resins. This process was repeated three times.

### 2.7. Characterization

**Fourier Transform Infrared Spectroscopy (FT-IR):** FT-IR spectra of samples were recorded on a Nicolet iS50 (Thermo Fisher Scientific, Waltham, MA, USA) instrument in the wavenumber range of 4000–400 cm^−1^ with a resolution of 4 cm^−1^ and 32 scans.

**Nuclear Magnetic Resonance Spectroscopy (NMR):** ^1^H NMR spectra were measured on a JNM-ECA 400 MHz spectrometer (JEOL Ltd., Musashino, Tokyo Metropolis, Japan) using DMSO-*d6* as the solvent and tetramethylsilane (TMS) as the internal standard.

**Differential Scanning Calorimetry (DSC):** The heat flow of the reaction between EP-VAN-HDA and MXDA (or MXDA-BGE, or MXDA-AGE) as a function of temperature was characterized on a NETZSCH DSC 214 instrument (NETZSCH, Selb, Bavaria, Germany). Measurements were performed at a heating rate of 5 °C/min from 25 °C to 160 °C under a nitrogen purge (50 mL/min).

**Thermogravimetric Analysis (TGA):** Approximately 10 mg of sample was used to characterize the thermal stability of epoxy resins on a TGA55 instrument (TA Instruments, New Castle, DE, USA). The measurement was conducted under nitrogen at a flow rate of 50 mL·min^−1^ over a temperature range of 100–800 °C.

**Dynamic Mechanical Analysis (DMA):** Samples of dimensions 15 mm (length) × 5 mm (width) × 2 mm (thickness) were cut. Dynamic mechanical properties were measured on a NETZSCH DMA 242 instrument (NETZSCH, Selb, Bavaria, Germany) in the temperature range of 30–150 °C at a heating rate of 5 °C·min^−1^, a frequency of 1 Hz, and an amplitude of 10 μm. The cross-linking density (*ν_e_*) of the epoxy resin was calculated using the following equation:

$$\nu_{e}=\frac{E^{\prime }}{3RT}$$

where $E^{\prime }$ is the storage modulus at $T_{g}+60$ °C, $T_{g}$ is the glass transition temperature, R is the gas constant, and T is the absolute temperature (in Kelvin) at $T_{g}+60$ °C.

**Mechanical Properties:** Tensile tests were performed on an Instron universal testing machine (Instron, Boston, MA, USA). The tensile specimens were rectangular (15 mm × 10 mm × 1 mm). The tensile test was conducted at a loading rate of 10 mm·min^−1^. Each sample was measured three times in parallel.

## 3. Results and Discussion

### 3.1. Structural Analysis of VAN-HDA and EP-VAN-HDA

**Figure 1:**
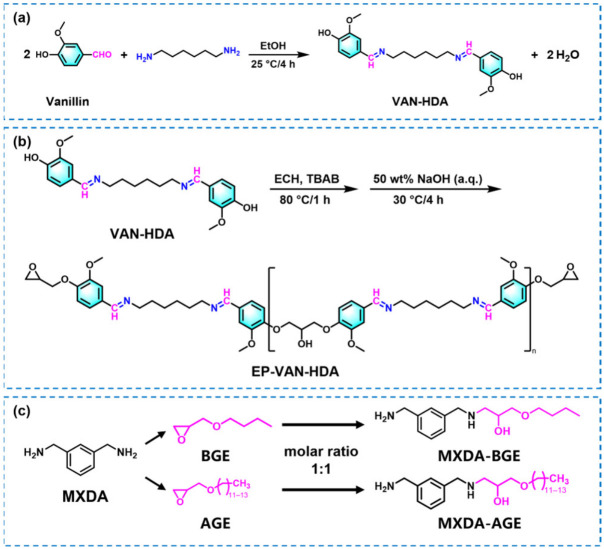
Schematic diagrams of the synthesis of (**a**) VAN-HDA, (**b**) EP-VAN-HDA, and (**c**) MXDA-BGE or MXDA-AGE.

**Figure 2:**
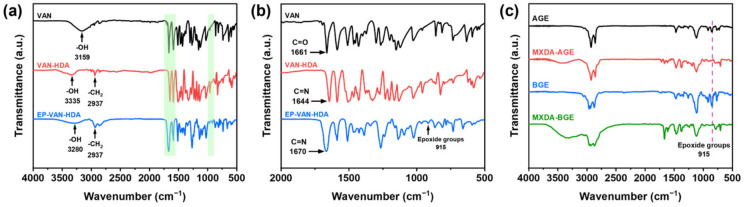
FT-IR spectra of (**a**) VAN-HDA, (**b**) EP-VAN-HDA, and (**c**) MXDA-BGE or MXDA-AGE.

As shown in Figure 1a–c, a vanillin-based epoxy resin containing Schiff base bonds (EP-VAN-HDA) and alkyl chain-grafted amine curing agents (MXDA-BGE or MXDA-AGE) were prepared, respectively. The chemical structures of EP-VAN-HDA and its precursor VAN-HDA were confirmed by FT-IR and ^1^H NMR. The FT-IR spectra of VAN-HDA and EP-VAN-HDA are shown in Figure 2a,b. Specifically, the green dashed area in Figure 2a and the magnified section at 2000–500 cm^−1^ in Figure 2b. The characteristic carbonyl peak at 1661 cm^−1^ disappeared. And the epoxy group peak at 915 cm^−1^ exhibited. New peaks corresponding to Schiff base bonds appeared at 1644 cm^−1^ and 1670 cm^−1^. The peak at 1644 cm^−1^ is assigned to the stretching vibration of the Schiff base bond conjugated with the benzene ring. The conjugation effect reduces the force constant of the Schiff base bond, shifting the absorption peak to a lower wavenumber. The peak at 1670 cm^−1^ corresponds to the Schiff base bonds in the liquid vanillin-based epoxy containing Schiff base. In this state, the Schiff base bonds are more likely to participate in intermolecular hydrogen bonding. This reduces the electron density on the nitrogen atom of the Schiff base bond. It alters the local chemical environment of the nitrogen atom. Consequently, this affects the vibrational frequency of the Schiff base bond. These results indicate that Schiff base bonds and epoxy groups were successfully introduced into the prepared EP-VAN-HDA. The successful synthesis of EP-VAN-HDA was further confirmed by ^1^H NMR spectroscopy, as shown in Figure S1a,b. Besides the epoxy groups, a proton peak corresponding to hydroxyl groups appeared near δ = 5.67 ppm. This hydroxyl peak originates from the polycondensation between VAN-HDA and ECH during the reaction. The polycondensation also generated oligomers, which caused broad peaks in the ^1^H NMR spectrum. During the synthesis of EP-VAN-HDA, when VAN-HDA reacted with excess epichlorohydrin, in addition to the target epoxy monomer, a small amount of linear oligomers was generated via side reactions between the phenolic hydroxyl groups of VAN-HDA and epoxy groups. Due to differences in molecular weight distribution and conformation, these oligomers broaden the peaks in the δ 3.5–4.5 ppm region of the ^1^H NMR spectrum [24]. From the integrated peak areas, the degree of polymerization (n) of EP-VAN-HDA was calculated to be 0.38, giving an average molecular weight of 664.01 g·mol^−1^. Based on the ^1^H NMR integration, the epoxy equivalent weight of the synthesized vanillin-based epoxy resin EP-VAN-HDA was calculated to be 332.0 g·eq^−1^, which is consistent with the measured value. Furthermore, FT-IR was used to confirm whether BGE or AGE successfully grafted onto MXDA. The results are shown in Figure 2c. The epoxy absorption peak at 915 cm^−1^ disappeared for both MXDA-BGE and MXDA-AGE, indicating that the reaction between the amino and epoxy groups occurred, and the alkyl chains were successfully grafted onto the MXDA molecule.

In summary, the linear-structured vanillin-based epoxy resin EP-VAN-HDA and the mono-grafted MXDA-BGE or MXDA-AGE were successfully synthesized. This provides data support for the subsequent calculation of the molar ratio between epoxy groups and active hydrogens during the curing process.

### 3.2. Curing Conditions for EP-VAN-HDA with MXDA, MXDA-BGE, and MXDA-AGE

**Figure 3:**
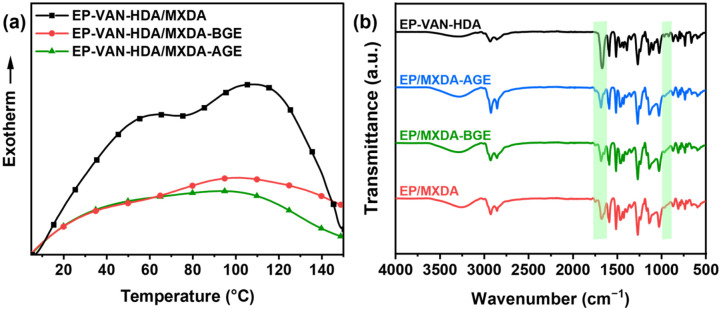
(**a**) DSC curves of the curing reactions of EP-VAN-HDA/MXDA, EP-VAN-HDA/MXDA-BGE, and EP-VAN-HDA/MXDA-AGE, (**b**) FT-IR spectra of EP-VAN-HDA/MXDA, EP-VAN-HDA/MXDA-BGE, and EP-VAN-HDA/MXDA-AGE.

The reactivity of EP-VAN-HDA with MXDA, MXDA-BGE, and MXDA-AGE was determined to further evaluate the curing temperatures of each system. Differential scanning calorimetry (DSC) was performed on the three precured samples. As shown in Figure 3a, the unmodified EP-VAN-HDA/MXDA curing system exhibited exothermic peaks at 64 °C and 106 °C. This is because the reaction between epoxy monomers and amine curing agents consists of the ring-opening reactions of primary and secondary amines with epoxy groups. Since primary amines have higher reactivity than secondary amines, two exothermic peaks appear. However, the EP-VAN-HDA/MXDA-BGE and EP-VAN-HDA/MXDA-AGE systems both showed exothermic peaks near 100 °C, and the full width at half maximum was proportional to the alkyl chain length. This is attributed to the higher molecular weight of MXDA-BGE and MXDA-AGE, which hinders the kinetics of the curing reaction, thereby requiring a higher curing temperature. To further confirm that the epoxy groups disappeared after curing, FT-IR characterization was performed. As shown in green dashed area at Figure 3b, after curing at 80 °C for 4 h, the characteristic absorption peak of epoxy groups (915 cm^−1^) completely disappeared but the characteristic absorption peak of the Schiff base (1670 cm^−1^) was retained in the FT-IR spectra of the three samples: EP-VAN-HDA/MXDA, EP-VAN-HDA/MXDA-BGE, and EP-VAN-HDA/MXDA-AGE. This indicates that the epoxy groups fully reacted with the active hydrogens of the amine curing agents, forming a cross-linked epoxy resin.

**Figure 4:**
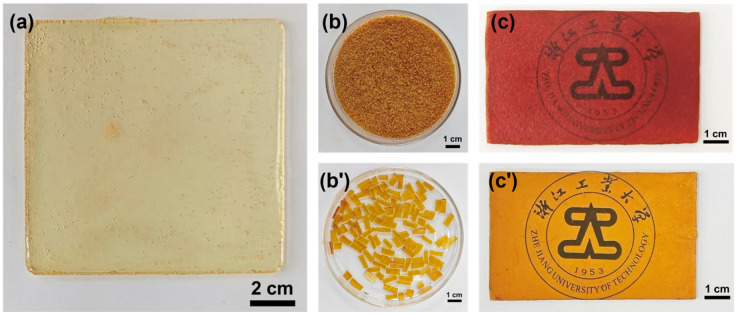
(**a**) Digital photos of EP-VAN-HDA/MXDA after pre-curing; Fragmented EP VAN HDA/MXDA specimens after curing at 80 °C (**b**,**c**) and 60 °C (**b′**,**c′**), alongside their corresponding hot-pressed samples.

However, during the curing of the vanillin-based epoxy resin, the viscosity is high, and the gelation rate is fast. As shown in Figure 4a, bubbles produced by stirring cannot be removed before a non-flowable gel forms. This greatly affects the mechanical properties of the vanillin-based epoxy resin. To eliminate bubbles during curing, the dynamic reversible Schiff base bonds in the prepared vanillin-based epoxy resin can be activated under high temperature and high pressure. This allows the cross-linked networks to reorganize and thus eliminate bubbles. Therefore, the pre-cured vanillin-based epoxy resin needs to be crushed and then re-molded by hot pressing using a flat vulcanizer. During hot pressing, the network reorganizes, and bubbles are removed.

Based on this, an attempt was first made to hot-press the EP-VAN-HDA/MXDA sample. This system has a higher average functionality and thus a higher cross-linking density. A high cross-linking density generally affects the exchange efficiency of dynamic covalent bonds. Therefore, hot-pressing a sample with high cross-linking density can verify the feasibility of this method. As shown in Figure 4b,c, the EP-VAN-HDA/MXDA system was pre-cured at 80 °C for 4 h. The obtained cured product containing bubbles was crushed into powder for hot pressing. After hot pressing, the sample showed disadvantages such as dark color, poor uniformity, and poor transparency.

To address this, the pre-curing temperature of EP-VAN-HDA/MXDA was adjusted to 60 °C. This temperature is close to the exothermic peak temperature of the reaction between primary amines and epoxy groups. Therefore, even if a non-flowable gel forms, its cross-linking density is still low, and further hot pressing can increase the cross-linking density. As shown in Figure 4b′,c′, after crushing and hot pressing the EP-VAN-HDA/MXDA sample pre-cured at 60 °C for 4 h, the obtained sample exhibited advantages such as little color change, high uniformity, and high transparency, which are better than those of the sample cured at 80 °C. In summary, after pre-curing the vanillin-based epoxy resin at 60 °C to obtain a gel-state product, further hot pressing reconstructs and forms complete epoxy cross-linked networks. This eliminates bubbles and achieves high mechanical properties.

### 3.3. Hot-Pressing Conditions for Cured EP-VAN-HDA/MXDA, EP-VAN-HDA/MXDA-BGE, and EP-VAN-HDA/MXDA-AGE

**Figure 5:**
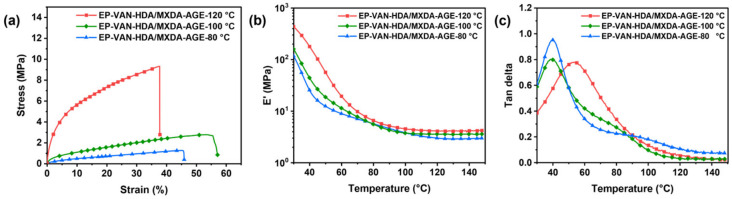
(**a**) Tensile stress–strain curves and (**b**,**c**) DMA curves of EP-VAN-HDA/MXDA-AGE after hot-pressing at different temperatures.

To determine the hot-pressing temperature for the pre-cured vanillin-based epoxy resin, the EP-VAN-HDA/MXDA-AGE cured product was selected to investigate the changes in mechanical properties at different hot-pressing temperatures. This is because the EP-VAN-HDA/MXDA-AGE cured product contains long alkyl chains. At a relatively low cross-linking density, the interactions between alkyl chains are weak, making it difficult to form a high-strength material. As the cross-linking density increases, the interactions between alkyl chains gradually become stronger, leading to a significant improvement in mechanical properties. As shown in Figure 5a, after hot-pressing at 80 °C and 100 °C for 1 h, the tensile strength of EP-VAN-HDA/MXDA-AGE increased from 1.2 MPa to 2.8 MPa, an increase of 133%. After hot-pressing at 120 °C for 1 h, the tensile strength further increased from 2.8 MPa to 9.3 MPa, an increase of 232%. Although the temperature difference was the same, the improvement in mechanical properties showed an exponential trend. This is mainly attributed to the increase in cross-linking density of EP-VAN-HDA/MXDA-AGE with increasing temperature. The alkyl dangling chains then undergo chain entanglement to form physical cross-linking points. With more alkyl chains participating in physical cross-linking, the mechanical properties of EP-VAN-HDA/MXDA-AGE increase significantly.

The thermomechanical properties of EP-VAN-HDA/MXDA-AGE samples processed at different hot-pressing temperatures were analyzed. As shown in Figure 5b, the sample processed at 120 °C exhibited a higher storage modulus, which corresponds to its higher tensile strength. Similarly, as shown in Figure 5c, the tanδ peak of the sample processed at 120 °C shifted to a higher temperature, indicating an increase in glass transition temperature. This is attributed to more complete cross-linked networks, which improve the mechanical properties. To further quantitatively analyze the degree of network perfection as a function of processing temperature, the cross-linking densities of the epoxy samples at different temperatures were calculated. The results are shown in Table S3. Compared to the sample processed at 80 °C, the cross-linking density of the sample processed at 100 °C increased by 27.6%; further increasing the temperature to 120 °C led to an additional increase of 9.5% relative to the 100 °C sample. Even though the increase in cross-linking density from 100 °C to 120 °C was small, the mechanical properties improved significantly. This is because DMA only measures the chemical cross-linking density, whereas the physical cross-linking between alkyl chains plays a major role in improving the mechanical properties of the EP-VAN-HDA/MXDA-AGE sample.

To better understand why such a drastic improvement in mechanical properties occurs only at 120 °C despite the modest change in chemical cross-linking density, chain mobility and network perfection were examined. At 100 °C, although above the glass transition, the motion of alkyl dangling chains remains restricted, limiting effective inter-chain entanglement. In contrast, at 120 °C, thermal energy significantly enhances alkyl chain mobility, allowing them to diffuse and entangle to form physical cross-linking points—analogous to temperature-dependent entanglement formation in thermoplastics. Notably, DMA may underestimate the contribution of physical entanglements at high temperatures (e.g., T_g_ + 60 °C) due to increased segmental motion. Supporting this interpretation, gel content measurements (Figures S2 and S3) show that samples processed below 120 °C contain soluble fractions, indicating incomplete network formation; the gel content increases from 82% at lower temperatures to 94% at 120 °C, confirming a more perfect cross-linked network with enhanced physical entanglements. Collectively, these results demonstrate that the substantial gain in mechanical properties at 120 °C arises primarily from physical entanglements rather than from the minor increase in chemical cross-linking density.

In summary, a hot-pressing temperature of 120 °C yields an EP-VAN-HDA/MXDA-AGE cured product with relatively good mechanical properties. Considering that Schiff bases are prone to side reactions such as cyclization and oxidation [25,26], the hot-pressing and reprocessing temperature was set to 120 °C, and the processing time was fixed at 1 h.

### 3.4. Mechanical and Thermal Properties of Cured EP-VAN-HDA/MXDA, EP-VAN-HDA/MXDA-BGE, and EP-VAN-HDA/MXDA-AGE

**Figure 6:**
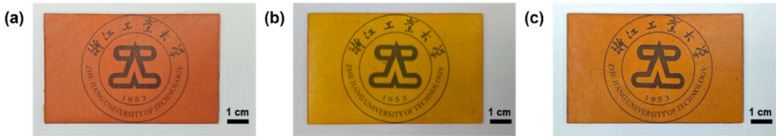
Digital photographs of (**a**) EP-VAN-HDA/MXDA, (**b**) EP-VAN-HDA/MXDA-BGE, and (**c**) EP-VAN-HDA/MXDA-AGE samples after hot-pressing at the temperature of 120 °C.

By optimizing the hot-pressing temperature, it was determined that a higher mechanical performance of the vanillin-based epoxy resin could be achieved at 120 °C, while the Schiff base bonds remained relatively stable at this temperature. Following the set hot-pressing temperature and time, EP-VAN-HDA/MXDA, EP-VAN-HDA/MXDA-BGE, and EP-VAN-HDA/MXDA-AGE were pre-cured at 60 °C for 4 h, then crushed and hot-pressed. Bubble-free and transparent vanillin-based epoxy resin cured products were obtained, as shown in Figure 6a–c.

**Figure 7:**
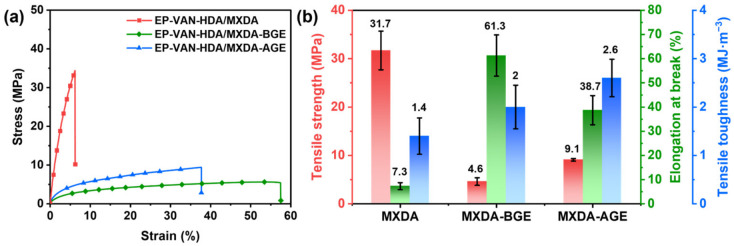
(**a**) Tensile stress–strain curves and (**b**) corresponding mechanical properties of EP-VAN-HDA/MXDA, EP-VAN-HDA/MXDA-BGE, and EP-VAN-HDA/MXDA-AGE.

Tensile tests were performed on the vanillin-based epoxy resins with different alkyl chain lengths to determine the effect of alkyl chain length on mechanical properties. The results are shown in Figure 7a,b and Table S4. After hot-pressing, EP-VAN-HDA/MXDA exhibited a relatively high mechanical strength of 31.7 MPa, but showed brittle fracture characteristics with an elongation at break of only 7.3%. Therefore, BGE or AGE was introduced to improve the toughness of the vanillin-based epoxy resin through its long alkyl chains. However, BGE with a shorter alkyl chain gave EP-VAN-HDA/MXDA-BGE a higher elongation at break, while the introduction of AGE with a longer alkyl chain reduced the elongation at break to 38.7%. This is because the alkyl chains exist as dangling chains in the cross-linked networks. Longer alkyl chains have stronger intermolecular interactions, which increase the strength of the corresponding physical cross-linking points and thus reduce the elongation at break. Another indicator of material toughness is tensile toughness, which is the integrated area under the stress–strain curve. This indicator better balances strength and flexibility, so that materials with higher tensile toughness also have relatively high tensile strength. The results show that EP-VAN-HDA/MXDA-AGE has higher tensile toughness than EP-VAN-HDA/MXDA-BGE. This is because its tensile strength is better than that of EP-VAN-HDA/MXDA-AGE, while it still has a certain elongation at break. This indicates that the introduction of longer alkyl chains can better balance strength and flexibility, resulting in a vanillin-based epoxy resin with relatively high toughness.

**Figure 8:**
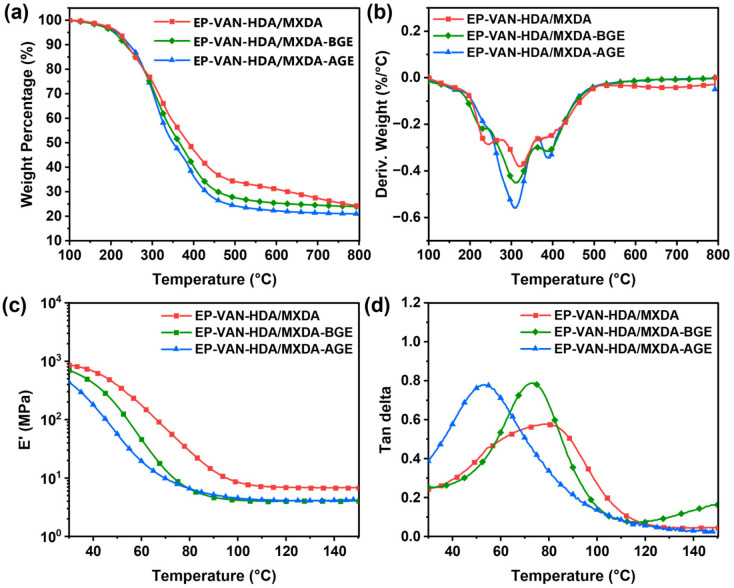
(**a**) TG curves, (**b**) DTG curves, and (**c**,**d**) DMA curves of EP-VAN-HDA/MXDA, EP-VAN-HDA/MXDA-BGE, and EP-VAN-HDA/MXDA-AGE.

Thermogravimetric analysis was performed to confirm whether the introduction of alkyl chains affects the thermal stability of the vanillin-based epoxy resin. The results are shown in Figure 8a,b and Table S5. The results indicate that although the introduction of alkyl chains affects the thermal decomposition temperatures (T_d5%_ and T_dmax_) and the final char yield (R_800_), the decreases in T_d5%_ and T_dmax_ are within 5%. This shows that the vanillin-based epoxy resins containing alkyl chains still retain good thermal stability because the alkyl chains are distributed as dangling chains within the cross-linked networks. On the basis of the original chemical cross-linked networks, physical cross-linking points formed by the aggregation of alkyl chains are also present, so the thermal stability of the final vanillin-based epoxy resin is almost unaffected. In addition, for the final char yield R_800_, EP-VAN-HDA/MXDA-AGE has a lower R_800_ than the other two epoxy resins. This is because EP-VAN-HDA/MXDA-AGE contains longer and higher proportion of alkyl chains. During thermal decomposition, the linear molecular chains are converted into small molecules by thermal cracking and escape, resulting in a relatively low char yield. Furthermore, according to the DTG curve shown in Figure 8b, EP-VAN-HDA/MXDA exhibits two thermal weight-loss peaks, with T_d1_ = 242.7 °C and T_d2_ = 324.1 °C, and T_d2_ is the maximum thermal weight loss peak of this epoxy resin. Based on the peak intensity, it can be considered that in the initial stage of thermal decomposition, the epoxy cross-linked network undergoes thermal cracking, and some of the resulting small molecules volatilize in advance, leading to a thermal weight-loss peak appearing at a relatively low temperature. For EP-VAN-HDA/MXDA-BGE and EP-VAN-HDA/MXDA-AGE, although two thermal weight-loss peaks also exist, the relatively weaker peak appears near ~390 °C. This is because these two vanillin-based epoxy resins contain alkyl dangling chains in their cross-linked networks. After the initial thermal cracking, the resulting small molecular components become entangled with the alkyl chains, which significantly increases their volatilization temperature, causing the weaker thermal weight-loss peak to appear around 390 °C. In summary, the similar T_d5%_ and T_dmax_ values indicate that the intrinsic thermal stability of the cross-linked networks is not significantly affected.

To determine the differences in dynamic mechanical properties, cross-linking density, and glass transition temperature of the vanillin-based epoxy resins with different alkyl chain lengths, DMA tests were performed. The results are shown in Figure 8c,d and Table S6. Because MXDA was modified with alkyl chains, the vanillin-based epoxy resins containing alkyl chains have a lower average functionality, and thus lower cross-linking density and glass transition temperature. However, since the molar ratios of glycidyl ethers with different alkyl chain lengths to MXDA were kept the same, the cross-linking densities of the obtained EP-VAN-HDA/MXDA-BGE and EP-VAN-HDA/MXDA-AGE samples were similar. Nevertheless, EP-VAN-HDA/MXDA-AGE has longer alkyl chains, so its glass transition temperature is lower than that of EP-VAN-HDA/MXDA-BGE. Although the introduction of alkyl chains reduces the cross-linking density and glass transition temperature of the vanillin-based epoxy resin, the storage modulus at temperatures below 40 °C shows little difference. This is because the alkyl chains are distributed as side chains in the epoxy cross-linked networks, preserving the rigidity of the network and providing high tensile toughness. Furthermore, as shown in Figure 8d, the tanδ peak intensities of EP-VAN-HDA/MXDA-BGE and EP-VAN-HDA/MXDA-AGE are both higher than those of the control resin without alkyl chains, indicating that the motion of the dangling alkyl chains increases the viscous dissipation of the molecular chains. In addition, the tanδ peak of EP-VAN-HDA/MXDA-AGE not only shifts to a lower temperature but also exhibits a larger full width at half maximum, suggesting a broader distribution of relaxation times in the system, which is consistent with its structural characteristics of having both chemical cross-linking points and physical aggregation points formed by the alkyl chains. In summary, the strategy of adjusting the cross-linking density by introducing dangling alkyl chains achieves retention of strength near room temperature and improvement of tensile toughness.

### 3.5. Reprocessing Performance of Cured EP-VAN-HDA/MXDA, EP-VAN-HDA/MXDA-BGE, and EP-VAN-HDA/MXDA-AGE

**Figure 9:**
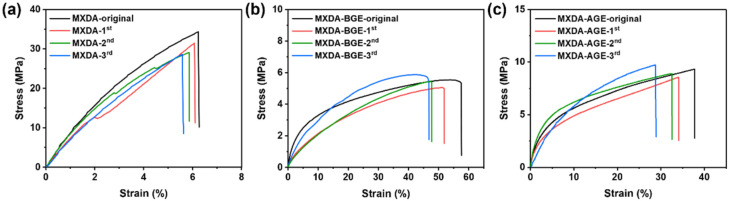
Tensile stress–strain curve of (**a**) EP-VAN-HDA/MXDA, (**b**) EP-VAN-HDA/MXDA-C_4_, and (**c**) EP-VAN-HDA/MXDA-C_12–14_ after three cycles of machining.

Since the three prepared vanillin-based epoxy resins with different alkyl chain lengths all possess rigid cross-linked networks formed by dynamic Schiff base bonds, they can be remolded under heating. To this end, the three resins were crushed and then hot-pressed at 120 °C for 1 h to obtain regenerated vanillin-based epoxy resins. As shown in Figure 9a–c, mechanical tests were performed on the reprocessed samples. The results show that all three resins still retain a certain tensile strength after three reprocessing cycles. Notably, the epoxy resins cured with MXDA-BGE or MXDA-AGE, which contain alkyl dangling chains, exhibited increased tensile strength after multiple reprocessing cycles. The main reason for this phenomenon is the synergistic effect between the rigid cross-linked networks and the alkyl dangling chains: first, some Schiff base bonds undergo self-cross-linking during reprocessing [26], and the resulting four-membered ring structures increase the chemical cross-linking density of the rigid network, thereby enhancing the tensile strength; second, the introduction of alkyl dangling chains regulates the chemical cross-linking density of the system, giving the vanillin-based epoxy resin higher chain segment mobility during hot pressing, thus making it easier to process and promoting topological rearrangement and network reconstruction; third, the alkyl dangling chains form reversible physical cross-linking points through physical entanglement during hot pressing, compensating for the loss of chemical cross-linking density caused by the introduction of the alkyl dangling chains and synergistically improving the mechanical strength together with the rigid networks. In summary, the synergistic effect of dynamic covalent bond self-cross-linking and alkyl dangling chain physical entanglement enables the vanillin-based epoxy resins containing alkyl chains to achieve no loss or even an increase in mechanical strength after multiple reprocessing cycles. Therefore, this synergistic regulation strategy effectively resolves the trade-off between strength and processing stability in reprocessable epoxy resins, providing a new approach for the design of high-performance, reprocessable epoxy resins.

To further verify the reversibility of cross-linking during reprocessing, gel fraction measurements were performed on the three resins after three reprocessing cycles. The samples were immersed in THF at room temperature for 7 days. As shown in Figures S4 and S5, all three reprocessed resins retained their sample integrity after immersion, and their gel fractions exceeded 90%. These results indicate that the powdered vanillin-based epoxy resins successfully reorganized into three-dimensional cross-linked networks via Schiff base exchange reactions during reprocessing, confirming the reversibility of the cross-linked networks.

**Figure 10:**
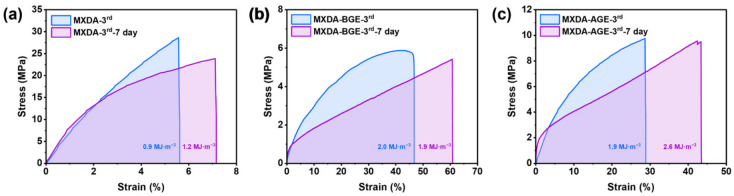
Tensile stress–strain curves of vanillin-based epoxy resins after three rounds of reprocessing, following 7 days of storage at room temperature and 50% humidity. (**a**) EP-VAN-HDA/MXDA-3rd, (**b**) EP-VAN-HDA/MXDA-BGE-3rd, (**c**) EP-VAN-HDA/MXDA-AGE-3rd.

In addition, to evaluate the short-term storage stability of the reprocessed vanillin-based epoxy resins, samples that had undergone three reprocessing cycles were stored for 7 days at room temperature and 50% relative humidity, followed by tensile testing. As shown in Figure 10a–c, all three resins retained relatively high tensile strength and elongation after this storage period. Specifically, compared to its counterpart before storage (Figure 9a), EP-VAN-HDA/MXDA-3rd-7 day (Figure 10a) exhibited a slight decrease in tensile strength, which may be attributed to partial hydrolysis or relaxation of the Schiff base network under humid conditions. In contrast, as shown in Figure 10b,c, EP-VAN-HDA/MXDA-BGE and EP-VAN-HDA/MXDA-AGE maintained tensile strengths comparable to those measured before storage, indicating that the presence of alkyl dangling chains helps mitigate humidity-induced degradation. This protective effect likely stems from increased hydrophobicity and enhanced chain mobility, which facilitate network rearrangement and reduce moisture absorption. Furthermore, the tensile toughness of all three samples was preserved or improved to some extent. Overall, the results in Figure 10 indicate that these three reprocessed vanillin-based epoxy resins exhibit satisfactory short-term storage stability under ambient conditions, supporting their potential for practical applications.

In summary, these results demonstrate that reprocessing enables the effective reorganization of the cross-linked networks while maintaining excellent short-term storage stability, especially for resins bearing alkyl dangling chains, highlighting their potential for sustainable applications.

## 4. Conclusions

In summary, by introducing rigid and reversible Schiff base bonds to construct a chemical cross-linking backbone and synergistically incorporating alkyl dangling chains, the epoxy resin is endowed with a reprocessable mechanism similar to that of thermoplastic elastomers. During multiple reprocessing cycles, the rigid cross-linked networks undergo network reconstruction through dynamic covalent bond exchange, while the alkyl dangling chains form reversible physical cross-linking points via physical entanglement, compensating for the loss of chemical cross-linking density. As a result, the mechanical properties are retained or even partially enhanced. By tuning the length of the alkyl dangling chains, an epoxy resin with moderate mechanical strength and elongation at break was obtained, and its tensile toughness was increased by 85.7% compared to the epoxy resin without dangling chains. Furthermore, although the introduction of alkyl dangling chains reduces the cross-linking density of the epoxy resin, its thermal stability and glass transition temperature are largely retained, with corresponding loss rates within 5% and 10%, respectively. By leveraging the physical entanglement of alkyl dangling chains during processing to avoid mechanical property loss, and the rigid cross-linked networks to provide basic strength and heat resistance, this study ultimately achieves high performance and reprocessability for thermosetting resins, taking epoxy resins as a representative example.

## Data Availability

The original contributions presented in this study are included in this. article/Supplementary Materials. Further inquiries can be directed to the corresponding authors.

## Supplementary Materials

The following supporting information can be downloaded at: https://www.mdpi.com/article/10.3390/polym18101226/s1, Table S1: Mass ratio for the graft modification reaction of MXDA with BGE or AGE; Table S2: Mass ratio for the curing reaction of EP-VAN-HDA with MXDA, MXDA-BGE, or MXDA-AGE; Table S3: Crosslinking density and T_g_ for EP-VAN-HDA/MXDA-AGE at different processing temperatures; Table S4: Mechanical Properties of EP-VAN-HDA/MXDA, EP-VAN-HDA/MXDA-BGE, and EP-VAN-HDA/MXDA-AGE; Table S5: Thermal Properties of EP-VAN-HDA/MXDA, EP-VAN-HDA/MXDA-BGE, and EP-VAN-HDA/MXDA-AGE; Table S6: Crosslinking density and T_g_ for EP-VAN-HDA/MXDA, EP-VAN-HDA/MXDA-BGE, and EP-VAN-HDA/MXDA-AGE; Figure S1: ^1^H NMR spectra of (a) VAN-HDA and (b) EP-VAN-HDA. Figure S2: Digital photographs of EP-VAN-HDA/MXDA-AGE prepared at different processing temperatures, before and after immersion in tetrahydrofuran at room temperature. (a) Before immersion; (b) After immersion. Figure S3: Gel content of EP-VAN-HDA/MXDA-AGE prepared at different processing temperatures after soaking in tetrahydrofuran at room temperature for 7 days. Figure S4: Digital photographs of vanillin-based epoxy resin after three rounds of reprocessing, taken before and after immersion in tetrahydrofuran at room temperature. (a) Before immersion; (b) After immersion. Figure S5: Gel content of vanillin-based epoxy resin after three rounds of reprocessing following a 7-day immersion in tetrahydrofuran at room temperature.

## References

[1] Zhang S., Liu T., Hao C., Mikkelsen A., Zhao B., Zhang J. (2020). Hempseed Oil-Based Covalent Adaptable Epoxy-Amine Network and Its Potential Use for Room-Temperature Curable Coatings. ACS Sustain. Chem. Eng..

[2] Eyann L., Fatah Muhamed Mukhtar M.A., Saad A.A., Jaafar M. (2025). Epoxy Molding Compounds for High-Performance Electronic Packaging: A Review on Recent Studies. Mater. Sci. Semicond. Process..

[3] Soutis C. (2005). Fibre Reinforced Composites in Aircraft Construction. Prog. Aerosp. Sci..

[4] Zhang S., Liu T., Hao C., Wang L., Han J., Liu H., Zhang J. (2018). Preparation of a Lignin-Based Vitrimer Material and Its Potential Use for Recoverable Adhesives. Green Chem..

[5] Yang J., Liu J., Liu W., Wang J., Tang T. (2015). Recycling of Carbon Fibre Reinforced Epoxy Resin Composites under Various Oxygen Concentrations in Nitrogen–Oxygen Atmosphere. J. Anal. Appl. Pyrol..

[6] Xing M., Li Z., Zheng G., Du Y., Chen C., Wang Y. (2021). Recycling of Carbon Fiber-Reinforced Epoxy Resin Composite via a Novel Acetic Acid Swelling Technology. Compos. Part B.

[7] Hahladakis J.N., Velis C.A., Weber R., Iacovidou E., Purnell P. (2018). An Overview of Chemical Additives Present in Plastics: Migration, Release, Fate and Environmental Impact during Their Use, Disposal and Recycling. J. Hazard. Mater..

[8] Saccani A., Manzi S., Lancellotti I., Lipparini L. (2019). Composites Obtained by Recycling Carbon Fibre/Epoxy Composite Wastes in Building Materials. Constr. Build. Mater..

[9] Liu Y., Meng L., Huang Y., Du J. (2004). Recycling of Carbon/Epoxy Composites. J. Appl. Polym. Sci..

[10] Montarnal D., Capelot M., Tournilhac F., Leibler L. (2011). Silica-Like Malleable Materials from Permanent Organic Networks. Science.

[11] Zych A., Tellers J., Bertolacci L., Ceseracciu L., Marini L., Mancini G., Athanassiou A. (2021). Biobased, Biodegradable, Self-Healing Boronic Ester Vitrimers from Epoxidized Soybean Oil Acrylate. ACS Appl. Polym. Mater..

[12] Yang S., Chu D., Dai S., Wang X., Du S., Zhang F., Ma S. (2025). Dynamic Ester-Enabled Recyclable Epoxy Adhesives from Glucose and Soybean Oil: Storage-Stable, High-Adhesion, and on-Demand Degradable. Macromolecules.

[13] Ruiz De Luzuriaga A., Martin R., Markaide N., Rekondo A., Cabañero G., Rodríguez J., Odriozola I. (2016). Epoxy Resin with Exchangeable Disulfide Crosslinks to Obtain Reprocessable, Repairable and Recyclable Fiber-Reinforced Thermoset Composites. Mater. Horiz..

[14] Ma S., Wei J., Jia Z., Yu T., Yuan W., Li Q., Wang S., You S., Liu R., Zhu J. (2019). Readily Recyclable, High-Performance Thermosetting Materials Based on a Lignin-Derived Spiro Diacetal Trigger. J. Mater. Chem. A.

[15] Xu S., Zhou L., Fei J., Ma M., He H., Shi Y., Zhu Y., Chen S., Wang X. (2025). Dynamic Schiff Base Bonds Enable Recyclable, High-Toughness Vanillin Epoxy Resins via Micro–Nano-Phase Engineering. ACS Appl. Polym. Mater..

[16] Zhao H., Wei X., Fang Y., Gao K., Yue T., Zhang L., Ganesan V., Meng F., Liu J. (2022). Molecular Dynamics Simulation of the Structural, Mechanical, and Reprocessing Properties of Vitrimers Based on a Dynamic Covalent Polymer Network. Macromolecules.

[17] Memon H., Wei Y., Zhang L., Jiang Q., Liu W. (2020). An Imine-Containing Epoxy Vitrimer with Versatile Recyclability and Its Application in Fully Recyclable Carbon Fiber Reinforced Composites. Compos. Sci. Technol..

[18] Xu X., Ma S., Wang S., Wu J., Li Q., Lu N., Liu Y., Yang J., Feng J., Zhu J. (2020). Dihydrazone-Based Dynamic Covalent Epoxy Networks with High Creep Resistance, Controlled Degradability, and Intrinsic Antibacterial Properties from Bioresources. J. Mater. Chem. A.

[19] Mo R., Song L., Hu J., Sheng X., Zhang X. (2020). An Acid-Degradable Biobased Epoxy-Imine Adaptable Network Polymer for the Fabrication of Responsive Structural Color Film. Polym. Chem..

[20] Chen M., Luo W., Lin S., Zheng B., Zhang H. (2023). Recyclable, Reprocessable, Self-Healing Elastomer-like Epoxy Vitrimer with Low Dielectric Permittivity and Its Closed-Loop Recyclable Carbon Fiber Reinforced Composite. Compos. Part B.

[21] Jin Y., Hu C., Wang Z., Xia Z., Li R., Shi S., Xu S., Yuan L. (2023). Bio-Based Reprocessable and Degradable Epoxy Resins via Inverse Vulcanization. ACS Sustain. Chem. Eng..

[22] Stajcic I., Veljkovic F., Petrovic M., Veličkovic S., Radojevic V., Vlahović B., Stajcic A. (2023). Impact- and Thermal-Resistant Epoxy Resin Toughened with Acacia Honey. Polymers.

[23] Bekeshev A., Mostovoy A., Shcherbakov A., Tastanova L., Akhmetova M., Apendina A., Orynbassar R., Lopukhova M. (2023). The Influence of Pristine and Aminoacetic Acid-Treated Aluminum Nitride on the Structure, Curing Processes, and Properties of Epoxy Nanocomposites. J. Compos. Sci..

[24] Fache M., Viola A., Auvergne R., Boutevin B., Caillol S. (2015). Biobased Epoxy Thermosets from Vanillin-Derived Oligomers. Eur. Polym. J..

[25] Ciaccia M., Di Stefano S. (2015). Mechanisms of Imine Exchange Reactions in Organic Solvents. Org. Biomol. Chem..

[26] Zhao X.-L., Zhang Z.-W., Li Y.-D., Du A.-K., Wu Y., Zeng J.-B. (2023). Topological Manipulation of Fully Biobased Poly(Epoxy Imine): From Thermoplastic Elastomers to Covalent Adaptable Networks and Permanently Cross-Linked Networks. ACS Sustain. Chem. Eng..

