# Supplementary material for: Mechanically Enhanced and Reprocessable Vanillin-Based Epoxy Resin via Synergistic Effect of Rigid Cross-Linked Networks and Alkyl Dangling Chains
Source: Polymers (Basel). 2026 May 17;18(10):1226. doi: 10.3390/polym18101226 (PMC13210534; doi:10.3390/polym18101226)
Supplement: Supplementary file 1 [file polymers-18-01226-s001.zip › polymers-4282287-supplementary.pdf]

Supplementary Material

Mechanically Enhanced and Reprocessable Vanillin-Based Epoxy Resin via Synergistic Effect of Rigid Crosslinked Network and Alkyl Dangling Chains

Likang Zhou [1], Songjie Xu [2,3], Junhao Fei [1], Meng Ma [2], Huiwen He [2], Yanqin Shi [2], Yulu Zhu [2], Si Chen [2,3,] and Xu Wang [2,*]*

1 College of Advanced Materials Engineering, Jiaxing Nanhu University, Jiaxing 314000, China.

2 College of Materials Science and Engineering, Zhejiang University of Technology, Hangzhou 310014, China.

3 Pinghu Institute of Advanced Materials, Zhejiang University of Technology, Jiaxing 314000, China.

* Correspondence: chensi@zjut.edu.cn (S.C.); wangxu@zjut.edu.cn (X.W.)

1. Proportions of the components in the MXDA alkylation modification and EP-VAN-HDA curing reactions

Table S1. Mass ratio for the graft modification reaction of MXDA with BGE or AGE.

Sample	Mass fraction (%)		
	MXDA	BGE	AGE
MXDA-BGE	51.1	48.9	-
MXDA-AGE	30.5	-	69.5

The mass fraction was calculated based on the molar ratio of the epoxy groups in BGE or AGE to MXDA;

The molar concentrations of epoxy groups in BGE and AGE are 7.7 mmol/g and 3.1 mmol/g, respectively (as measured according to GB/T 1677—2008).

Table S2. Mass ratio for the curing reaction of EP-VAN-HDA with MXDA, MXDA-BGE, or MXDA-AGE.

Sample	Mass fraction (%)			
	EP-VAN-HDA	MXDA	MXDA-BGE	MXDA-AGE
EP-VAN-HDA/MXDA	90.7	9.3	-	-
EP-VAN-HDA/MXDA-BGE	78.8	-	21.2	-
EP-VAN-HDA/MXDA-AGE	68.5	-	-	31.5

The mass fraction was calculated based on the molar ratio of the epoxy groups in EP-VAN-HDA to the active hydrogen in MXDA and its derivatives;

The molar concentration of epoxy groups in EP-VAN-HDA is 3.0 mmol/g (as measured according to GB/T 1677—2008);

The molar concentrations of reactive hydrogen for MXDA, MXDA-BGE, and MXDA-AGE were calculated as 29.4 mmol/g, 11.2 mmol/g, and 6.5 mmol/g, respectively.

2. Structural Characterization of VAN-HDA and EP-VAN-HDA

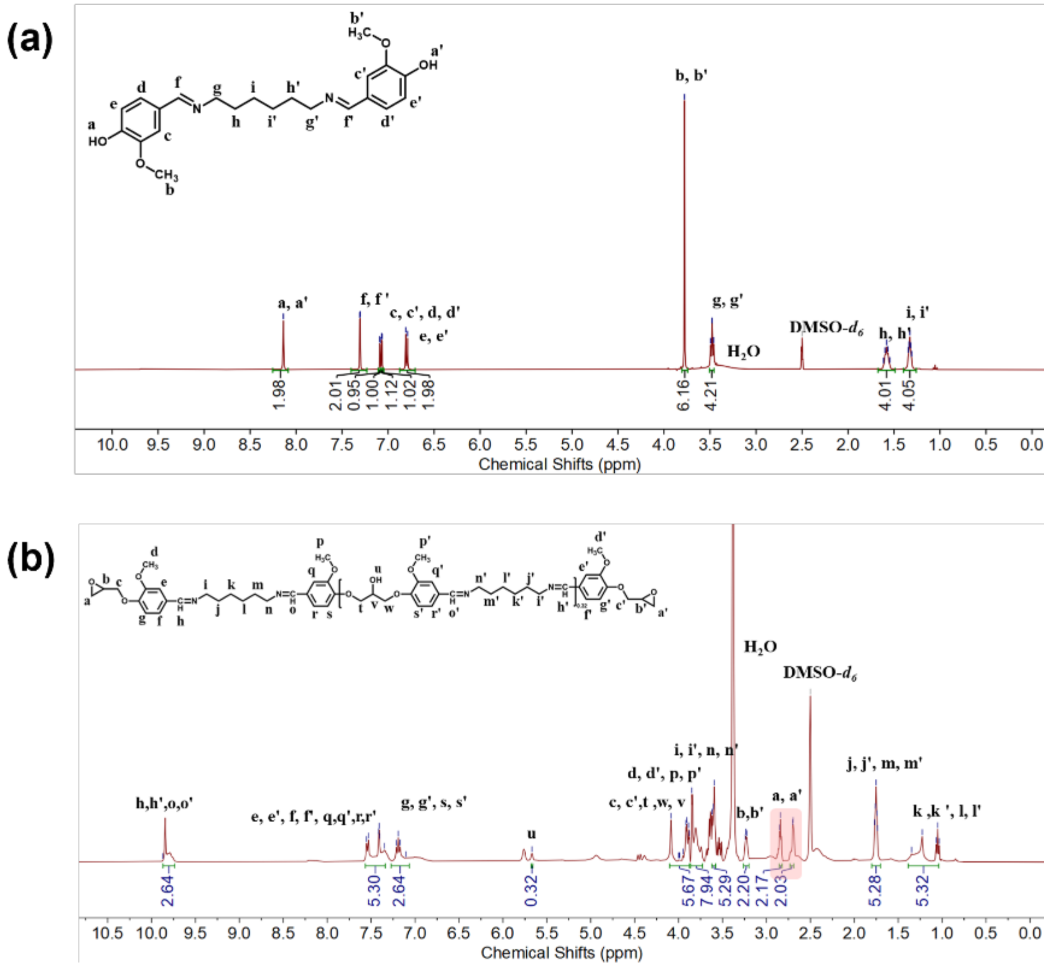

Figure S1. ^{1}H NMR spectra of (**a**) VAN-HDA and (**b**) EP-VAN-HDA.

3. The Effect of Hot-Pressing Temperature on the Cross-Linked Network Properties of EP-VAN-HDA/MXDA-AGE

Table S3. Crosslinking density and T_g for EP-VAN-HDA/MXDA-AGE at different processing temperatures.

Sample	E_r' (MPa)	ν_e (mol·m^{-3})	T_g (°C)
EP-VAN-HDA/MXDA-AGE-120 °C	4.2	436	53
EP-VAN-HDA/MXDA-AGE-100 °C	3.7	398	40
EP-VAN-HDA/MXDA-AGE-80 °C	2.9	312	39

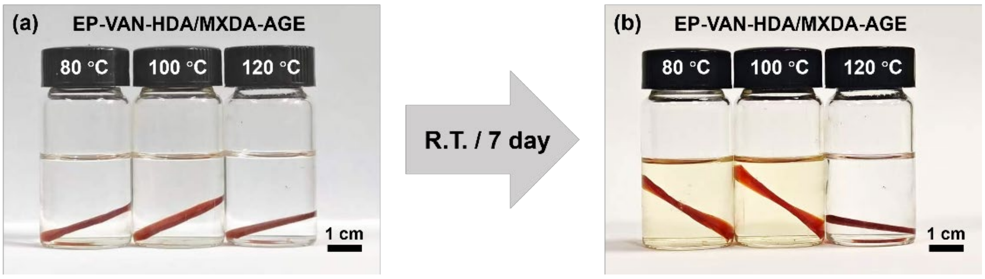

Figure S2. Digital photographs of EP-VAN-HDA/MXDA-AGE prepared at different processing temperatures, before and after immersion in tetrahydrofuran at room temperature. (a) Before immersion; (b) After immersion.

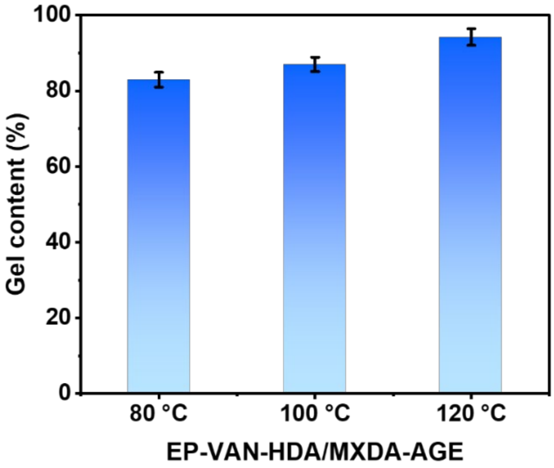

Figure S3. Gel content of EP-VAN-HDA/MXDA-AGE prepared at different processing temperatures after soaking in tetrahydrofuran at room temperature for 7 days.

4. Vanillin-Based Epoxy Resins with Different Alkyl Dangling Chain Lengths: Mechanical Properties and Thermomechanical Performance

Table S4. Mechanical Properties of EP-VAN-HDA/MXDA, EP-VAN-HDA/MXDA-BGE, and EP-VAN-HDA /MXDA-AGE.

Sample	Tensile strength (MPa)	Elongation at break (%)	Tensile toughness (MJ·m-3)
EP-VAN-HDA/MXDA	31.7 ± 4.0	7.3 ± 1.4	1.4 ± 0.4
EP-VAN-HDA/MXDA-BGE	4.6 ± 0.8	61.3 ± 8.5	2.0 ± 0.5
EP-VAN-HDA/MXDA-AGE	9.1 ± 0.3	38.7 ± 6.0	2.6 ± 0.4

Table S5. Thermal Properties of EP-VAN-HDA/MXDA, EP-VAN-HDA/MXDA-BGE, and EP-VAN-HDA /MXDA-AGE.

Sample	$T_{d5\%}$ (°C)	T_{dmax} (°C)	R_{800} (%)
EP-VAN-HDA/MXDA	218.4	324.1	24.3
EP-VAN-HDA/MXDA-BGE	207.3	312.2	24.0
EP-VAN-HDA/MXDA-AGE	213.6	310.0	21.0

Table S6. Crosslinking density and T_g for EP-VAN-HDA/MXDA, EP-VAN-HDA/MXDA-BGE, and EP-VAN-HDA/MXDA-AGE.

Sample	E_r' (MPa)	ν_e (mol·m^{-3})	T_g (°C)
EP-VAN-HDA/MXDA	6.9	668	81
EP-VAN-HDA/MXDA-BGE	4.1	408	73
EP-VAN-HDA/MXDA-AGE	4.2	436	53

5. Cross-linking Degree of Vanillin-Based Epoxy Resins After Reprocessing

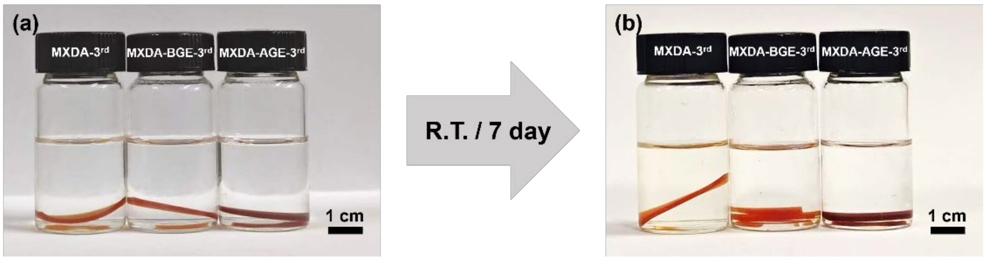

Figure S4. Digital photographs of vanillin-based epoxy resin after three rounds of reprocessing, taken before and after immersion in tetrahydrofuran at room temperature. (a) Before immersion; (b) After immersion.

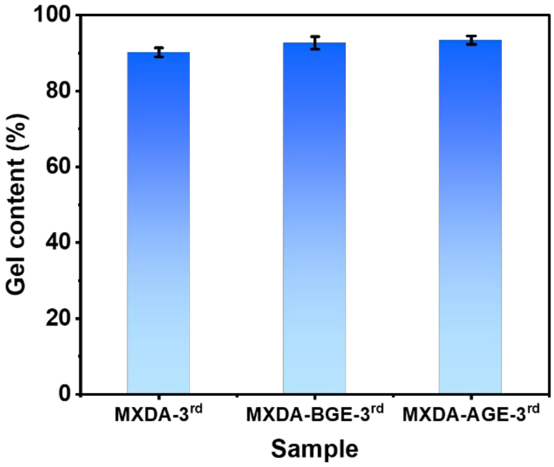

Figure S5. Gel content of vanillin-based epoxy resin after three rounds of reprocessing following a 7-day immersion in tetrahydrofuran at room temperature.
